# Polymorphism in interferon alpha/beta receptor contributes to glucocorticoid response and outcome of ARDS and COVID-19

**DOI:** 10.1186/s13054-023-04388-8

**Published:** 2023-03-16

**Authors:** Juho Jalkanen, Sofia Khan, Kati Elima, Teppo Huttunen, Ning Wang, Maija Hollmén, Laura L. Elo, Sirpa Jalkanen

**Affiliations:** 1 Faron Pharmaceuticals, Turku, Finland; 2 Turku Bioscience Centre, University of Turku and Åbo Akademi University, Turku, Finland; 3 InFLAMES Flagship, University of Turku and Åbo Akademi University, Turku, Finland; 4 Institute of Biomedicine, University of Turku, Turku, Finland; 5 Estimates, Turku, Finland; 6 MediCity Research Laboratory, University of Turku, Tykistökatu 6, 20520 Turku, Finland

**Keywords:** Type I interferons, COVID-19, ARDS, Glucocorticoids

## Abstract

**Background:**

The use of glucocorticoids has given contradictory results for treating acute respiratory distress syndrome (ARDS). The use of intravenous Interferon beta (IFN β) for the treatment of ARDS was recently tested in a phase III ARDS trial (INTEREST), in which more than half of the patients simultaneously received glucocorticoids. Trial results showed deleterious effects of glucocorticoids when administered together with IFN β, and therefore, we aimed at finding the reason behind this.

**Methods:**

We first sequenced the genes encoding the IFN α/β receptor of the patients, who participated in the INTEREST study (ClinicalTrials.gov Identifier: NCT02622724, November 24, 2015) in which the patients were randomized to receive an intravenous injection of IFN β-1a (144 patients) or placebo (152 patients). Genetic background was analyzed against clinical outcome, concomitant medication, and pro-inflammatory cytokine levels. Thereafter, we tested the influence of the genetic background on IFN α/β receptor expression in lung organ cultures and whether, it has any effect on transcription factors STAT1 and STAT2 involved in IFN signaling.

**Results:**

We found a novel disease association of a SNP rs9984273, which is situated in the interferon α/β receptor subunit 2 (IFNAR2) gene in an area corresponding to a binding motif of the glucocorticoid receptor (GR). The minor allele of SNP rs9984273 associates with higher IFNAR expression, more rapid decrease of IFN γ and interleukin-6 (IL-6) levels and better outcome in IFN β treated patients with ARDS, while the major allele associates with a poor outcome especially under concomitant IFN β and glucocorticoid treatment. Moreover, the minor allele of rs9984273 associates with a less severe form of coronavirus diseases (COVID-19) according to the COVID-19 Host Genetics Initiative database.

**Conclusions:**

The distribution of this SNP within clinical study arms may explain the contradictory results of multiple ARDS studies and outcomes in COVID-19 concerning type I IFN signaling and glucocorticoids.

**Graphical abstract:**

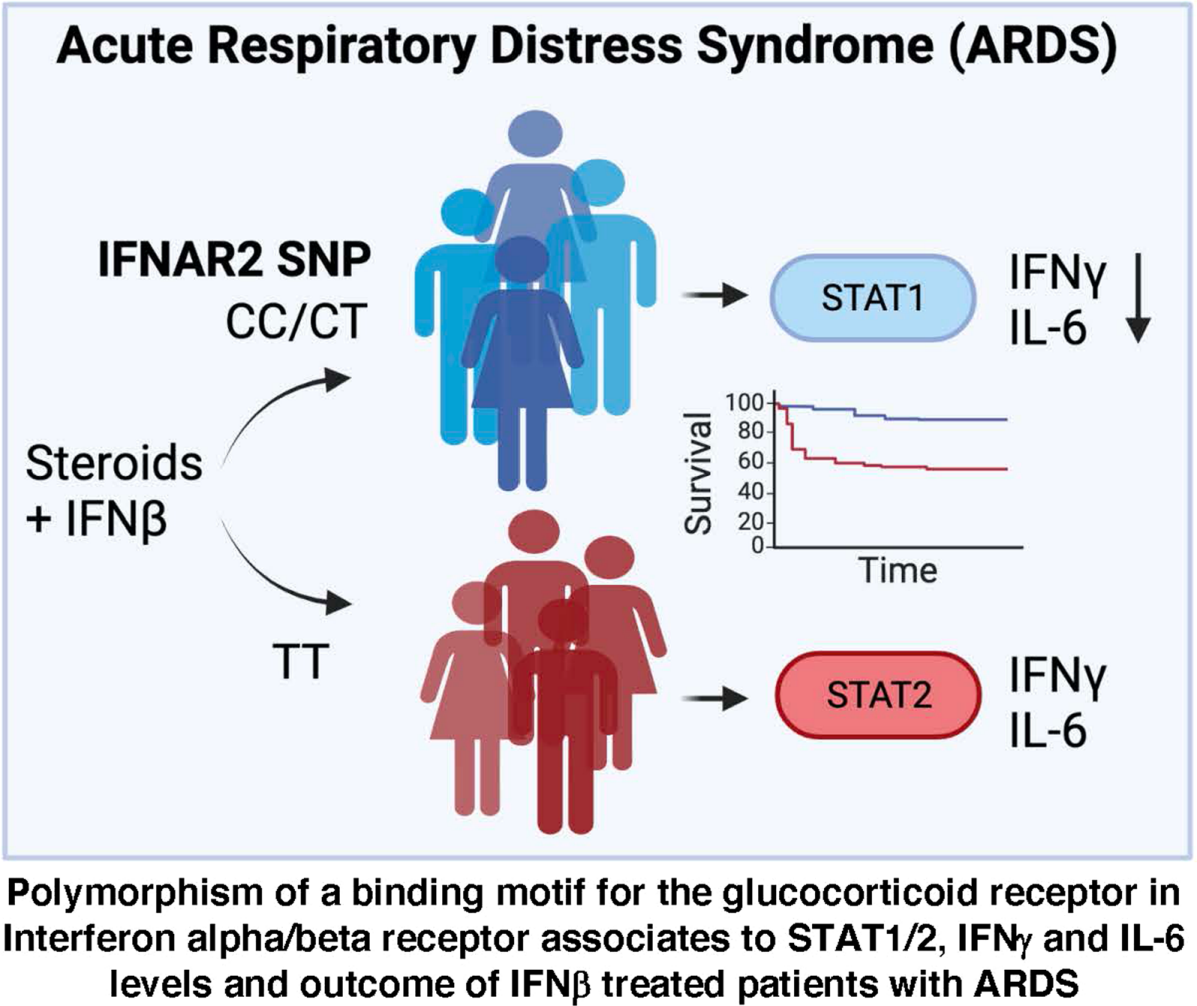

**Supplementary Information:**

The online version contains supplementary material available at 10.1186/s13054-023-04388-8.

## Introduction

**I**nterferon (IFN) and cortisol are our natural responses to viral insult and stress, respectively, and the inability to summon either is deadly [1–3]. It appears that these natural responses need to happen in a sequential manner, since endogenous cortisol and exogenous glucocorticoids block IFN signaling [4–6] and are known to be harmful if given early in viral diseases [7]. For decades in the clinical management of ARDS, sepsis, and now COVID-19, glucocorticoids have given controversial results and their use still remains an ongoing debate [7–13]. The current SARS-CoV2 pandemic has demonstrated that late in the disease when excessive inflammation and poor oxygenation prevail glucocorticoids can be beneficial [14]. In addition to glucocorticoids, Type I IFNs are also considered as viable treatments for COVID-19, Middle East respiratory syndrome (MERS), and ARDS, but contrary to glucocorticoids IFNs should be administered early [15–17]. Given late in the disease course of COVID-19 or in non-COVID-19 ARDS, the combination of intravenous IFN β and glucocorticoids has even shown to be harmful [5, 18]. Hence, we hypothesize that nature has never intended these two fundamental pathways to be “on” at the same time. In our work, we identified a novel disease association for the SNP rs9984273, which is situated in the gene of IFNAR2. Its location corresponds to a binding motif for the glucocorticoid receptor (GR). It appears that this SNP, at least to a certain extent, blocks glucocorticoid interference with type I IFN signaling, thereby enabling us to take advantage of these two pathways. The minor allele of the SNP is associated with a milder disease in COVID-19 and better survival in patients with ARDS when glucocorticoids are used together with IFN β. Here, we elucidate our findings with the help of clinical studies and the publicly available COVID-19 GWAS Task Force data.

## Methods

### Clinical study populations

The clinical study population has been extensively characterized and reported [18]. Shortly, The INTEREST trial ClinicalTrials.gov Identifier: NCT02622724 was a multi-center, randomized, double-blind, parallel-group trial conducted at 74 intensive care units in eight European countries (from December 2015 to December 2017) that included 296 adults with moderate-to-severe ARDS. Patients were randomized to receive an intravenous injection of 10 μg IFN β-1a (144 patients) or placebo (152 patients) for six consecutive days. We illustrate the possible interaction between rs9984273 and IFN β-1a and impact on survival by presenting Kaplan–Meyer survival curves until 1-year follow-up for both study arms with and without concomitant presence of SNP rs9984273. A logistic regression model including age, gender, ARDS severity, Acute Physiology and Chronic Health Evaluation (APACHE) II score, concomitant glucocorticoid use with IFN β, and SNP status was used to evaluate independent factors related to 28-day mortality.

The association of rs9984273 with survival is reported using the entire study population with genetic data available. For the IFN γ analyses, only patients with ARDS due to pneumonia or sepsis or pulmonary origin and using glucocorticoids were included as they best resemble COVID-19 ARDS patients. There were 75 of those patients with an available genetic consent and samples available. Daily serum samples were drawn for cytokine measurements. Pro-inflammatory cytokine levels were analyzed using Bio-Plex Cytokine Assay by Bio-Rad Laboratories (Hercules, USA). A repeated measurements ANCOVA (RMANCOVA) for change from the baseline in log scale (adjusted for baseline log-transformed value) was used to compare daily IFN γ and IL-6 levels between the patients according to the SNP rs9984273 status.

### Lung specimens

Lung specimen from 14 different individuals was obtained by postsurgical resection of anonymous lung tissue (typically for cancer resections). The lung sections from lung regions having normal macro- and microscopic appearance were used. Several small pieces were cut from all samples and 5–6 pieces/well/condition were cultured for 4 days in 24 well plates containing 1 ml of RPMI medium (supplemented with 10% fetal calf serum, 4 mM L-glutamine, 100 U/ml penicillin, and 100 μg/ml streptomycin), IFN β (1000 UI/ml FP1201; Faron Pharmaceuticals), or placebo with and without hydrocortisone (40 µg/ml, Solucortef, Pfizer). After culturing, all pieces in each well were collected and frozen in OCT. All 14 samples were used for staining IFNAR1/IFNAR2 and 5 CT and 5 TT samples for STAT1, pSTAT1, STAT2, pSTAT2, and CD73 stainings.

DNA was extracted from 3 to 5 frozen lung tissue sections (20 µm) with NucleoSpin DNA Rapid Lyse Kit (Macherey–Nagel). TaqMan SNP genotyping assays C_2443264_10 and C_11354003_30 (Thermo Fisher Scientific) for rs9984273 and rs2236757, respectively, were used in genotyping. The reactions were performed as in TaqMan SNP genotyping manuals (ThermoFisher). The plate was run on a QuantStudio 3 Real-Time PCR machine (ThermoFisher) with a run protocol for SNP genotyping as per manual. The results were analyzed using the Applied Biosytems® analysis modules for genotyping in Thermo Fisher Cloud computing platform (ThermoFisher Scientific).

### Immunohistochemistry

Five-µm-thick frozen sections were cut from lung pieces and fixed with acetone for 5 min. Thereafter, the endogenic peroxidase was blocked using Bloxall Blocking solution (Vector laboratories) for 15 min at room temperature RT. The first-stage antibodies were against alpha and beta chains (IFNAR1 and IFNAR2) of the IFN α/β receptor (St John's Laboratory STJ112765 and LS-Bio LS-B13369, respectively). The anti-alpha chain antibody was used 1:2000 and 1:5000, anti-beta chain 1:1000, anti-STAT1 (1:400, 9175S), anti-pSTAT1 (1:100, 9167S), anti-STAT2 (1:200, 72604S), and anti-pSTAT2 (1:50, 88410S) all from Cell Signaling. Rabbit Ig (1 µg/ml) was used as a negative control. The secondary step was performed with Vectastain Elite ABC rabbit IgG kit (Vector laboratories) and diaminobenzidine was used as a chromogen.

Two different concentrations of the antibody against the alpha chain of the IFN α/β receptor were used to better recognize the differences between the samples. The staining intensity was blindly scored from 0 to 3 and the mean intensity obtained by these two concentrations was used as the score for the alpha chain; mean of this and that obtained for the beta chain intensity (0 to 3) is shown for IFNAR. The intensity of the STAT stainings was semi-quantitatively scored from 0 to 3 both at the whole cell level and separately in nuclei of positive cell types. CD73 was detected with a monoclonal anti-CD73 antibody (4G4) 10 µg/ml followed by Alexa Fluor488 conjugated goat anti-mouse IgG (Invitrogen). A negative class matched monoclonal antibody was used as a control. Number of positive vessels were counted and the staining intensity was scored from 0 to 4. On average of 20 fields/sample at 200× magnification were evaluated. All samples were treated in the similar way and analyzed blindly without knowledge of the patient identity.

### MX1 response

PBMCs were isolated from 9 mL of EDTA blood by Ficoll gradient centrifugation. The cells were plated 0.5 × 10^6^ cells/well in 96-well U-bottom plates (Sarstedt) and treated ± hydrocortisone 20 µg/mL (Solucortef, Pfeizer) in human plasma overnight. The next day, 1000 IU/mL of IFN-beta (Rebif) was added on the cells and incubated for another 48 h. Thereafter, RNA was extracted with the NucleoSpin RNA kit (Macherey–Nagel, Dueren, Germany) according to the manufacturer’s protocol. For qPCR assays, the conversion of RNA to cDNA was done with SuperScript VILO cDNA Synthesis Kit (Thermo Fisher Scientific, Espoo, Finland), followed by qPCR using the TaqMan Gene Expression Assays (Thermo Fisher Scientific, Espoo, Finland) for MX1 (Hs00895608_m1) and GAPDH (Hs02758991_g1; control gene). The reactions were run using the Applied Biosystems' Quant Studio 3 Real-Time PCR System (Thermo Fisher Scientific). The target mRNA levels were normalized to GAPDH and a fold change of relative expression from the appropriate unstimulated control was calculated using the Applied Biosystems® analysis modules in Thermo Fisher Cloud computing platform (Thermo Fisher Scientific, Espoo, Finland).

### Genetic analyses

The AmpliSeq® custom panel was used to sequence *NT5E (*= *CD73), IFNAR1* and *IFNAR2*. The panel was designed to cover the whole gene region (exons, introns, and untranslated regions (UTRs)) and 3 kb up- and down-stream. Paired-end sequencing with 2 × 300-bp read length was conducted with an Illumina MiSeq® instrument using v3 sequencing chemistry. The quality of the sequencing data was high, and only minor trimming was done to remove low quality bases from the data (< 1% of the data). The alignment and variant calling were conducted with the BaseSpace data analysis tool (https://basespace.illumina.com, Illumina). Differences in SNP rs9984273 genotype distribution between deceased and alive ARDS patients were tested using allelic chi-square test using the PLINK software [19]. Chi-square test was also used to assess the association between the carriers of the minor alleles of rs9984273 and rs2236757 and mortality.

### Binding site analysis

We investigated putative binding sites of GR within the SNP rs9984273 region utilizing the Transfac database (version 2020.3) [20] and Encode ChIP-seq data [21]. The Match tool in Transfac was used to find matches between the SNP rs9984273 region and the position weight matrices in the Transfac database. The quality of a match between the query region and the matrix was assessed by a matrix similarity score. The HaploReg tool [22] was used to find matches between the SNP rs9984273 region and the position weight matrices derived from the Encode ChIP-seq data [21]. Only matches that passed the stringent threshold of *P* < 4^–8^ reported by the tool were considered.

### COVID-19 host genetics initiative database analyses

The COVID-19 Host Genetics Initiative (COVID19-hg) GWAS meta-analyses rounds 4 and 6 summary results were used to identify the association of the SNP rs9984273 with disease severity [23]. The meta-analysis across multiple cohorts was performed with fixed effects inverse variance weighting and adjusted for age, sex, and genetic ancestry principal components. Cochran's Q heterogeneity test was used to examine heterogeneity among the cohorts. SNPs with minor allele frequency less than 0.001 and an imputation quality score (INFO) less than 0.6 were filtered out from each individual study before the meta-analysis. For SNP pruning, the European sub-cohort of the 1000 Genomes phase 3 was used. We inspected the rs9984273 risk association in the available subgroups of very severe respiratory confirmed covid, hospitalized covid, and covid, as compared to not hospitalized covid or general population.

### Statistics

Several statistical methods such as repeated measures ANCOVA, multivariate Cox model, logistic regression analysis, Cochran's Q heterogeneity test, Chi-square test, two-way ANOVA with Šídák's multiple comparisons test, and two-tailed Mann–Whitney U-test were used. Their use is indicated in the text and/or figure legends. A *P* value of less than 0.05 was considered statistically significant.

## Results

### Discovery of rs9984273 in clinical trials investigating intravenous IFN β-1a for the treatment of ARDS

We have performed two significant clinical trials investigating the use of intravenous IFN β-1a for the treatment of ARDS [18, 24]. The rationale for performing these trials was in the ability of intravenous IFN β to increase the expression of 5’-ectonucleotidase, CD73 on lung endothelium. CD73 is a key protective molecule and the rate limiting enzyme in converting pro-inflammatory adenosine monophosphate (AMP) into anti-inflammatory adenosine [25]. The first open-label Phase I/II study investigating intravenous IFN β-1a for ARDS produced remarkable results, showing ARDS mortality of only 8% [24]. The trial was small, consisting of only 37 ARDS patients in the active arm, but it led us to perform a substantially larger placebo controlled trial of 296 ARDS patients [18]. In this randomized clinical trial, intravenous IFN β showed no benefit over placebo in the entire study population. However, further laboratory analyses revealed that glucocorticoids block IFN signaling and the upregulation of CD73 in the lung endothelium and increased mortality in the active arm of the study [5]. Patients (*n* = 66) who did not receive glucocorticoids with IFN β had 28-day mortality of 10.6%, while patients (*n* = 78) who did receive glucocorticoids with IFN β had 28-day mortality of 39.7%. The deleterious effect of glucocorticoids administered together with intravenous IFN β was evident despite adjusting to disease severity and the likelihood of receiving glucocorticoid treatment in propensity matched multivariable regression analyses [5].

**Fig. 1:**
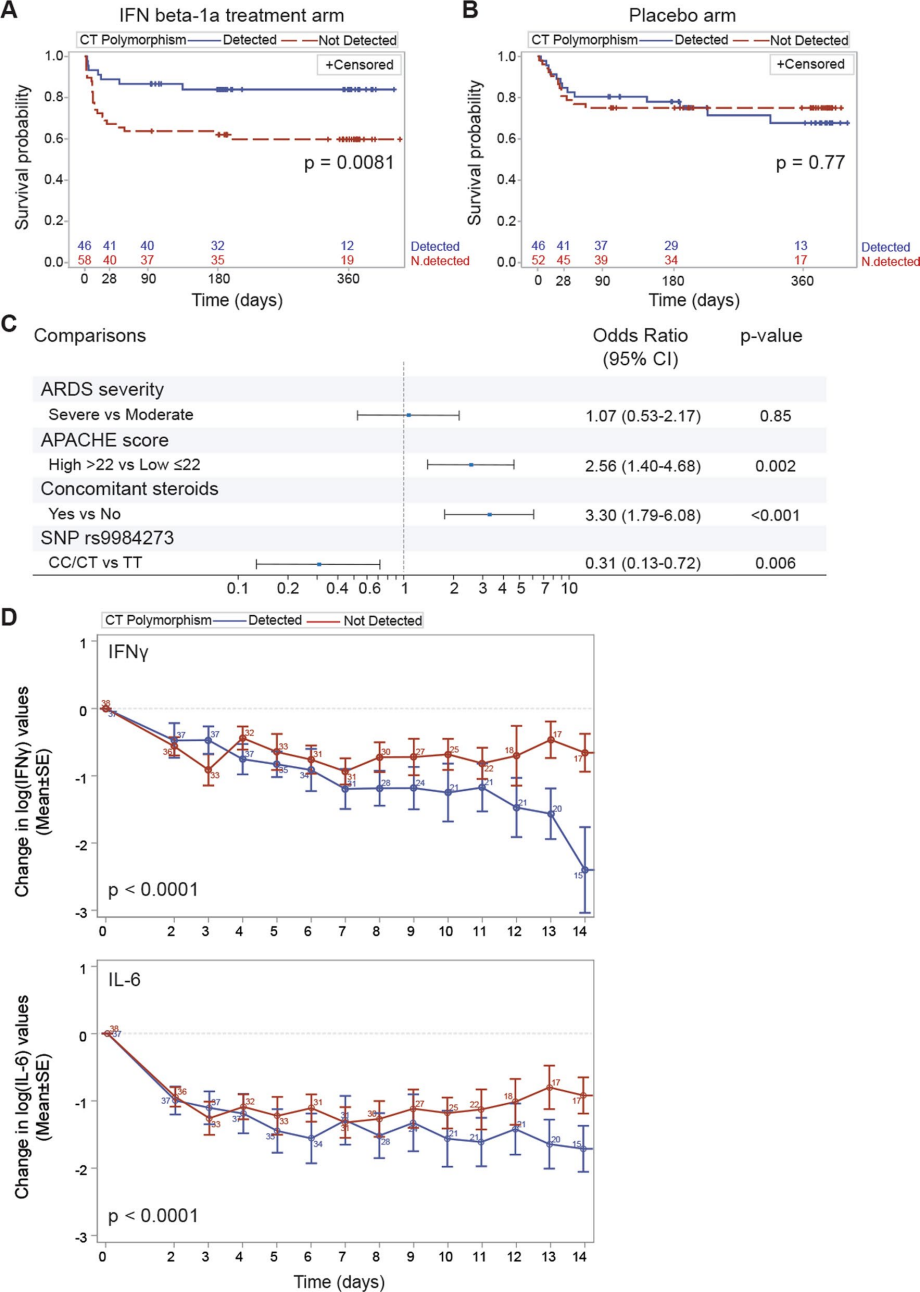
The effect of the rs9984273 genotype on mortality and cytokine levels in the INTEREST trial investigating intravenous IFN β-1a for ARDS treatment. A genetic sample was available from 202/296 (104/144 in the IFN β-1a treatment arm and 98/152 in the placebo arm) of the study subjects. Number of the subjects at risk is indicated on the x- axis. **A** The rs9984273 minor allele C (CC or CT) was significantly associated with survival in patients compared to those who were homozygous with the major allele (TT). **B** A similar association was not observed in the placebo arm. **C** Further adjusted logistic regression analysis for Day 28 mortality encompassing the entire Phase III study population (both active and placebo arms) revealed that the use of glucocorticoids had the strongest association with mortality. **D** The logarithmic change in serum IFN γ and IL-6 levels according to the genetic background of rs9984273 in patients of the INTEREST trial with ARDS of pulmonary origin. Patients with the minor allele (CT or CC) had more rapid decrease in serum IFN γ and IL-6 levels toward normal when glucocorticoids were administered than the patients homozygous with the major allele (TT) in rs9984273. Numbers of the patients at each time point are indicated

Further, pre-defined targeted resequencing and genetic analyses of entire gene regions were performed for genes encoding CD73 (*NT5E*) and IFN α/β receptor alpha and beta chains (*IFNAR1* and *IFNAR2*). In the initial analyses, the SNP associations with the IFN β-1a treatment response were inspected for all the SNPs in the sequenced regions and for the statistically significant associations (*P* < 0.05) with mortality was inspected. Out of these analyses, the minor C allele found with sequencing (rs9984273) was shown to significantly associate with decreased mortality in the IFN β treatment arm when compared to those patients having the major allele T. Patients with ARDS carrying the minor C allele had day-28 mortality of only 10.9% (vs 31.0% without minor C allele) when treated with IFN β. Figure 1A, 1B shows the survival curves up to day 360 for active and placebo arm by SNP status (*n* = 202 with the SNP status available). As can be seen, similar phenomenon was not witnessed in the placebo arm. When testing for the interaction using multivariate Cox model adjusted for Acute Physiology and Chronic Health Evaluation (APACHE) II score and concomitant glucocorticoid use, the interaction between the treatment group and SNP status was significant (*p* = 0.046). Furthermore, day 28 mortality was analyzed using multivariate logistic regression in the full population (*n* = 296). In this analysis, concomitant use of glucocorticoids with IFN β-1a was associated with increased mortality (OR 3.30; 95% CI 1.79–6.08; *P* < 0.001), while the presence of the minor allele C in rs9984273 was associated with lower mortality at day 28 (OR 0.31; 95% CI 0.13–0.72; *P* = 0.006) (Fig. 1C). There was no significant fluctuation in the effect of the polymorphism on day 28 mortality between countries (*p* = 0.7462 for polymorphism country interaction, the forest plot in Additional file 1: Fig. S1). Thus, the use of glucocorticoids and the presence of the minor allele in this SNP associate to the outcome of intravenous IFN β treatment in patients with ARDS.

The polymorphism affected differently the outcome of men and women in the INTEREST trial. Majority of the patients (70%) in the trial were male. 42.4% of men and 51.4% of women had CC/CT. Mortality of women was 11.8% for TT and 8.3% for CC/CT patients; mortality of men was 28.9% for TT patients and 12.5% for CC/CT patients. Thus, TT increases risks of death only in men, OR 2.85, *p* = 0.028, while it is not seen among the women, OR 1.47, *p* = 0.63 for an unknown reason.

Contribution of the SNP rs9984273 polymorphism was further investigated by a retrospective analysis, in which we performed focused genetic analyses of IFNAR2 on the earlier Phase I/II study population. Phase I/II and III study populations were comparable according to demographics and disease severity; however, we identified one major difference. In the Phase I/II study 40% of the study subjects receiving IFN β received overlapping glucocorticoids, but 73% of these patients had the rs9984273 the minor C allele. In contrast, in the phase III study 54% of the patients received glucocorticoids, but only 46% of the subjects receiving overlapping glucocorticoids with IFN β-1a carried the minor C allele. Hence, the rs9984273 C allele was highly enriched in the Phase I/II study population compared to the Phase III study, and more patients received glucocorticoids in the Phase III than in the Phase I/II study, thus suggesting a possible explanation for the difference between mortality in the studies.

### Rs9984273 associates with IFN γ and IL-6 production in patients receiving glucocorticoid treatment

The SNP rs9984273 locates in the 3’ untranslated region (3’-UTR) of the IFNAR2 gene (chr21:33,262,760 (GRCh38)). To investigate potential binding sites of GR in the region, we utilized position weight matrices compiled from individual genomic sites from the Transfac database (Transfac matrix table, Release 2020.3) or derived from the Encode ChIP-seq data for GR [21]. The Transfac-based results showed that the SNP resides in a predicted transcription factor binding site of GR (Transfac matrix similarity score 0.864, chr21:33,262,746–33,262,761). Furthermore, the SNP was predicted to alter a GR binding site on the basis of the ChIP-seq data (GR_known3 from [21]).

Following our in silico evaluations, we next wanted to determine, whether rs9984273 was associated with the immune status of the patients when glucocorticoids were administered. The Phase III ARDS trial (INTEREST) was optimal to analyze this effect as it had cytokine profiling of the patients for the first 14 days. IFN γ and IL-6 were chosen as indicators of immune activation as they are also associated with poor outcome in ARDS and COVID-19 [24, 26]. Only those patients with ARDS of pulmonary origin (pneumonia or pulmonary sepsis) were chosen for these analyses to best resemble ARDS of COVID origin and adjust for a heterogenic etiology of an all-comer ARDS trial, e.g., excluding aspiration. We found high interindividual variations in IFN γ and IL-6 levels and the values were not statistically significantly different between the patients homozygous with the major T allele and those with the minor allele C (CC or CT) in rs9984273 at the beginning, day 0 (IFN γ: 19.2 ± 38 pg/ml for CC/CT and 24 ± 40.5 pg/ml for TT; IL-6: 75.9 ± 275.3 pg/ml for CC/CT and 300.6 ± 1178.5 pg/ml for TT), but after day 7 IFN γ and IL-6 levels of the patients with the CC/CT genotype start to decrease back to normal faster than in TT patients (Fig. 1D). We hypothesize that administering patients with glucocorticoids is not harmful, if they possess at least one copy of the minor allele despite receiving simultaneous IFN β; however, in the patients under glucocorticoid treatment, the major allele TT is associated with higher levels of IFN γ and IL-6 (RMANCOVA, *P* < 0.0001), and the two anti-inflammatory agents, glucocorticoids and IFN β, become harmful. IL-6 and IFN γ values are presented separately according to patients’ mortality status on Day 28 in order to account of lost values due to death (Additional file 1: Fig. S2). Cytokine associations remain similar according to the genetic background despite clinical outcome.

### Rs9984273 contributes to the expression of IFNAR together with rs2236757

**Fig. 2:**
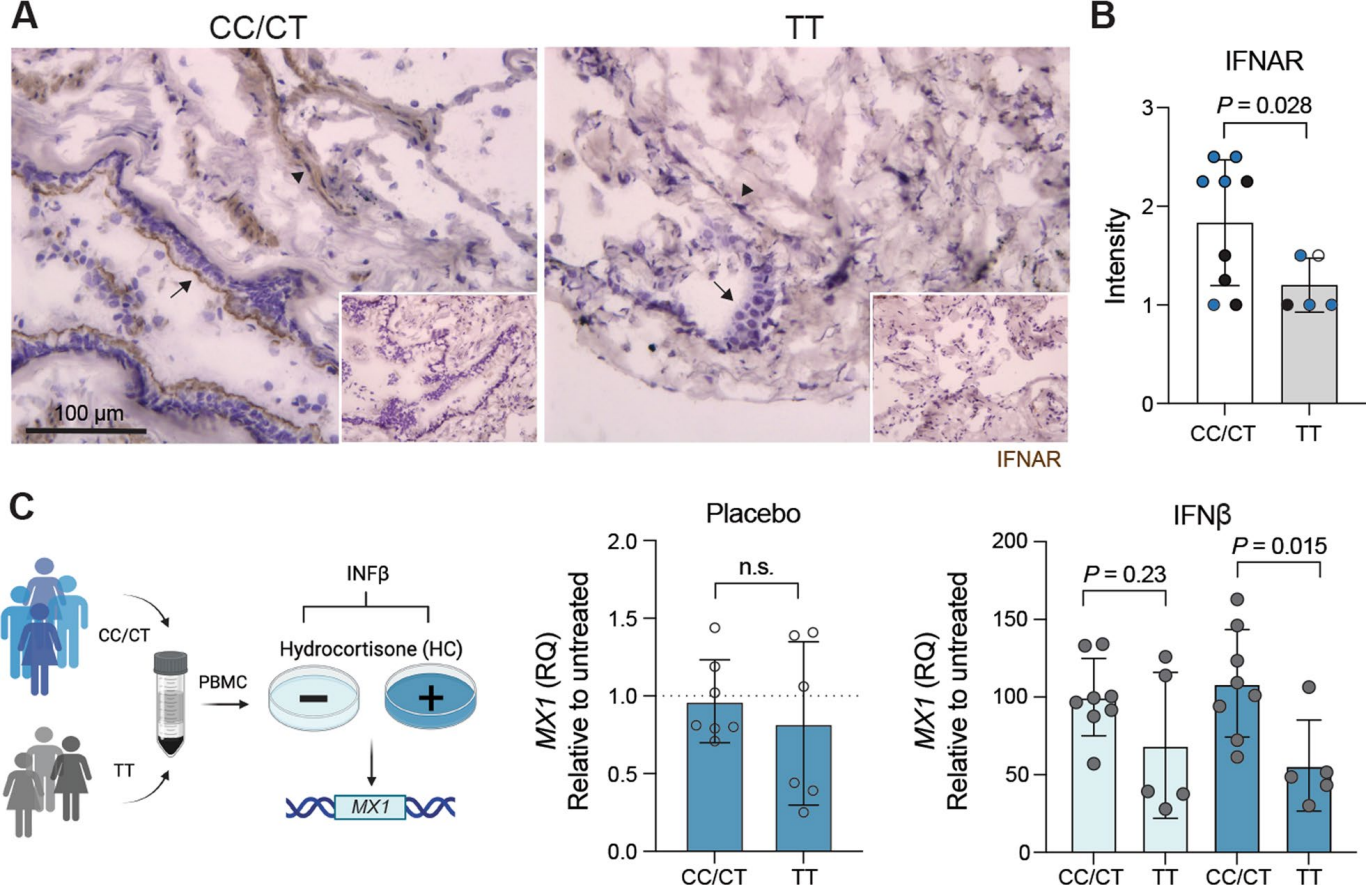
Rs9984273 is associated with the expression of IFNAR and regulation of *MX1* response. **A** Illustrative examples of IFNAR expression in lung specimens from patients with a CT (left panel) and a TT (right panel) genotype. Arrows indicate the alveolar epithelium and arrowheads the vessels. Stained negative controls are shown in the insets. **B** Combined expression intensity scores of rs9984273 (CC/CT and TT) and rs2236757 (AG, AA and GG) in lung specimens (*n* = 9 and 5, respectively). The single CC sample was pooled with CT samples for statistics between CC/CT and TT expressors. Color codes for rs2236757: AG blue, GG black, AA empty. Statistics: Mann–Whitney U-test, two-tailed. **C** *MX1* response. Peripheral blood mononuclear cells (PBMC) were isolated from healthy donors having CC/CT (*n* = 8) or TT (*n* = 5) and interferon response was measured with (dark blue) or without (light blue) hydrocortisone (HC) by *MX1* upregulation. The graphs show fold change (RQ) compared to the untreated sample (placebo without HC). Significance was calculated using unpaired *t* test with Welch’s correction

After the discovery of rs9984273, and recognizing its role in the outcome of the previous clinical ARDS studies, we then investigated, whether this polymorphism contributes to the expression of the IFNAR (composed of IFNAR1 and IFNAR2 subunits) by immunohistochemistry using antibodies against IFNAR subunits of the receptor in surgical lung specimens from 14 patients. In this material, five samples had the major allele TT, 8 samples CT, and 1 CC for rs9984273. The primary antibody was titrated to the level, at which the alveolar epithelium and vasculature still remained strongly positive in a subset of the samples. The scoring was done without any knowledge of the genotype. The results clearly show that all high expressors of IFNAR were CT and overall the CT/CC group had statistically significantly higher IFNAR level than the TT group. However, four out of nine samples from the CC/CT group show medium level of expression, indicating that other factors are involved in regulating IFNAR expression. Therefore, we decided to analyze another SNP, rs2236757 in IFNAR2 that has been found to contribute to disease severity of COVID-19 [27]. Eighty percent (4/5) of the highest IFNAR expressors had both rs2236757 A and rs9984273 C alleles, whereas three out of four medium expressors had rs2236757 G with rs9984273 C (Fig. 2B). Further, the A allele of rs2236757 alone was not enough for the high IFNAR expression as 80% (4/5) of low IFNAR expressors with rs9984273 TT had rs2236757 A. In summary, 8/9 of medium/low expressors did not have both rs2236757 A and rs9984273 C alleles (*p* = 0.005, Fisher’s exact test). Thus, A and C alleles of these SNPs together significantly contribute to the high expression of IFNAR in our lung specimens.

We also analyzed the number of CD73 positive vessels and expression level of CD73 in lung specimens incubated for 4 days with IFN β. There were no differences in the number of CD73 positive vessels/mm^2^ (2.6 ± 1.4 for CC/CT and 2.5 ± 0.8 for TT), but the staining intensity of CD73 was significantly higher in samples of CC/CT than TT patients (3.2 ± 0.4 and 2.2 ± 0.8, respectively; *p* = 0.04). Moreover, we collected peripheral blood mononuclear cells from CC/CT and TT individuals and measured *MX1* (a key mediator of the interferon-induced antiviral response against a wide range of viruses) after IFN β treatment. CC/CT individuals have significantly better response than TT individuals to IFN β measured as *MX1* increase suggesting better antiviral response (Fig. 2C).

### CT and TT patients display different features in IFN β-induced STAT1 and STAT2 signaling and responsiveness to glucocorticoids

**Fig. 3:**
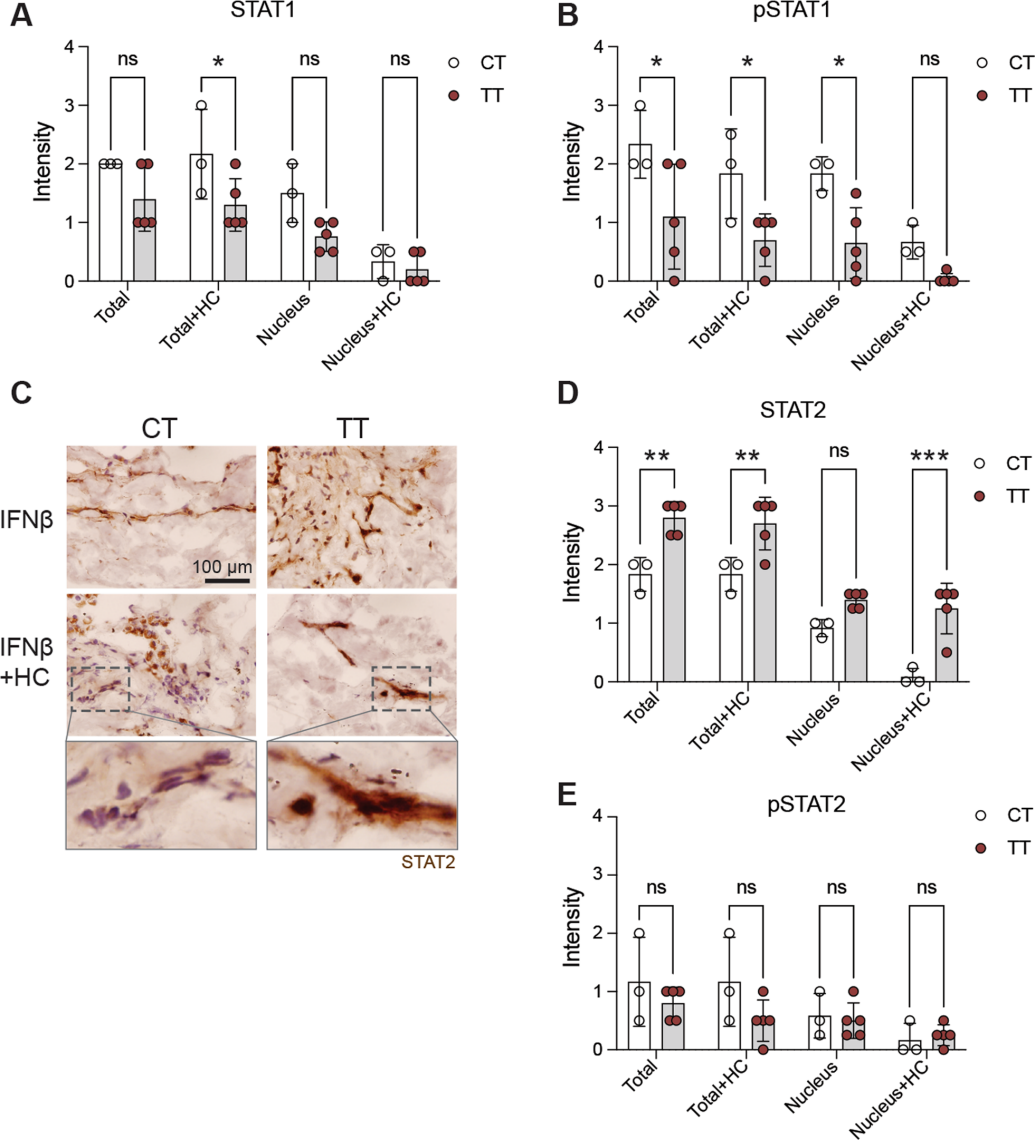
Patients homozygous for the major allele of rs9984273 (TT) have higher STAT2 expression in the lungs than the heterozygous patients with the minor allele (CT) and glucocorticoids do not inhibit its nuclear expression in TT patients.** A** STAT1 expression in the lung after a 4-day culture in the presence of IFN β with or without hydrocortisone (HC). **B** pSTAT1 expression in the same specimens as in A. **C** Example photomicrographs showing higher STAT2 expression in a TT patient than in a CT patient and the effect of HC on its nuclear translocation. Most STAT2 remains in the cytoplasm of the CT patients, whereas nuclear expression is prominent in the TT patient. Indicated insets are shown in the bottom row. **D** Combined results of all patients noting that two CT samples are excluded in the data as the patients were already under glucocorticoid treatment at the time of sample acquisition. **E** pSTAT2 expression in the same samples as in A, B and D. Ns, not significant; *P < 0.05; **P < 0.01; and ***P < 0.001. Statistics: two-way ANOVA with Šídák's multiple comparisons test

As the immune status and IFNAR expression are at least partially under the control of rs9984273 and the formation of the signal transducer and activator of transcription 1/2 and interferon regulatory factor 9 (STAT1/STAT2/IRF9) complex and its translocation into the nucleus is required to trigger type I IFN responsive genes, we next examined STAT1, phosphorylated STAT1 (pSTAT1), STAT2 and pSTAT2 expression in the lung specimens cultured in the presence of IFN β with or without hydrocortisone (HC) [4, 5]. We found that TT patients tended to have lower total STAT1 expression and statistically significantly less pSTAT1 than the CT patients and HC inhibited its nuclear location (Fig. 3A, B). In contrast, TT patients had significantly more STAT2 than the CT patients and HC did not decrease its nuclear expression like in CT patients (Fig. 3C, D), while no statistical differences in pSTAT2 were observed (Fig. 3 E). Examples of pSTAT1 and 2 staining are shown in Additional file 1: Fig. S3. For the STAT-analyses, we were limited being able to only include three out of five CT patients to the combined data shown in Fig. 3, as two sample donors were already on corticosteroids at the time of taking the lung specimen. In these two specimens, already on glucocorticoids, IFN β could not induce efficient nuclear localization of STAT1 and STAT2, further demonstrating the clinical role of glucocorticoids in this phenomenon.

### Rs9984273 is associated with COVID-19 hospitalization

Adhering to our recent clinical and laboratory findings, and the outbreak of the SARS-CoV2, we also explored the role of rs9984273 in COVID-19 through publicly available databases. After all, without a doubt both endogenous and exogenous type I IFNs and steroids play a fundamental role in disease progression and survival concerning COVID-19. Recent genome-wide analyses of COVID-19 patients have revealed that SNP rs2236757 in IFNAR2 has a significant impact on disease severity [27], but the functional role of these findings is yet to be explained and the contribution to signaling remains to be revealed. Until now, the importance of rs9984273 has not been recognized. Therefore, using the COVID-19 Host Genetics Initiative database, we explored whether there was an association between rs9984273 and disease severity (Table 1). We discovered that the presence of the minor allele of rs9984273 was associated with less hospitalization for COVID-19 (OR 0.96; 95%CI 0.93–1.00; *P* = 0.035), when comparing hospitalized to non-hospitalized COVID-19 patients. Hospitalized patients with COVID-19 also differed from the general public in the rs9984273 genotype distribution (OR 0.96; 95%CI 0.93–0.98; *P* = 0.001). Comparing the patient group with the most severe disease (very severe confirmed respiratory COVID-19) to the general public, the rs9984273 polymorphism showed a statistically significant risk association with the minor allele being associated with a lower risk of severe disease (OR 0.93; 95%CI 0.89–0.98; *P* = 0.006). The number of cohorts involved in the individual comparisons varied from 3 to 60 with no sign of heterogeneity between the cohorts (*P* > 0.3).

**Table 1:** Associations of rs9984273 C allele* to disease severity of COVID-19 in meta-analyses

Phenotype of cases and controls (database release)	Number of cases with genotype information	Number of controls with genotype information	Odds Ratio (95% confidence interval)	P value	Number of studies	P value from Cochran's Q heterogeneity test
Very severe respiratory confirmed covid vs. not hospitalized covid (release 4)	269**	688*	0.69 (0.46–1.02)	0.065	3	0.469
Very severe respiratory confirmed covid vs. general population (release 6)	6321	678,513	0.93 (0.89–0.98)	0.006	19	0.759
Hospitalized covid vs. not hospitalized covid (release 6)	13,582	66,291	0.96 (0.93–1.00)	0.035	20	0.313
Hospitalized covid vs. general population (release 6)	19,876	1,450,704	0.96 (0.93–0.98)	0.001	33	0.291
Covid vs. general population (release 6)	95,644	1,752,242	0.99 (0.98–1.00)	0.048	60	0.318

*The patients with CC/CT vs TT were compared

**Total number of cases and controls. Subjects involved in the calculation were 679 (distribution of cases and controls used in the calculation was not given in the original data)

We also tested, whether carrying the minor alleles of rs9984273 C and rs2236757 A, correlated with mortality in our ARDS cohort. We found that patients possessing the minor alleles for both genotypes have significantly decreased mortality than those homozygous with the major allele for both genotypes. The lower mortality among the minor allele carriers (*n* = 51) compared to non-carriers (*n* = 41) was seen at days 28, 90, 180, and 360, with 28-day mortality of 10% vs. 27% (*P* = 0.03), 90-day mortality of 16% vs. 37% (*P* = 0.02), 180-day mortality of 18% vs. 39% (*P* = 0.02), and 360-day mortality of 20% vs. 39% (*P* = 0.04), respectively. Although rs2236757 in IFNAR2 is associated with severe COVID-19 infection and low IFNAR expression to life-threatening disease [27], rs2236757 and rs9984273 are only in a mild linkage disequilibrium with (*r*^2^ = 0.19, D’ = 0.99) in the European population. Nevertheless, they may still significantly cooperate and regulate the expression of IFNAR as four out of five patients possessing both minor alleles had high levels of IFNAR expression.

## Discussion

**Fig. 4:**
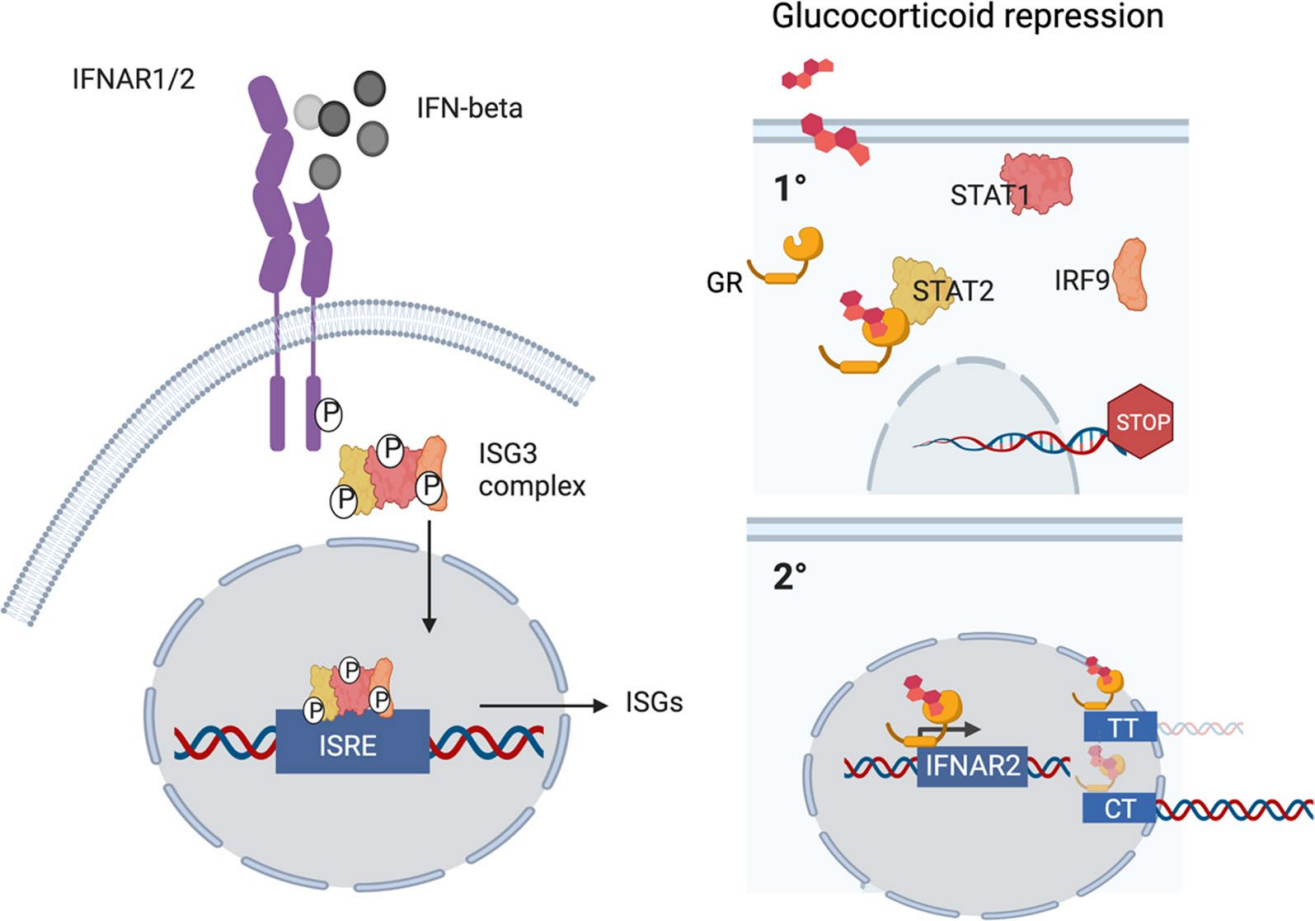
A schematic presentation regarding the mechanisms of action of glucocorticoids. In normal conditions, IFN β binding to its receptor triggers the formation of the ISG3 complex consisting of Stat1, Stat2, and IRF9. This complex then trans-locates from the cytoplasm to the nucleus where it binds to the interferon-stimulated response element (ISRE) in hundreds of interferon-stimulated genes (ISG) leading to the production of interferon-stimulated proteins. Glucocorticoids have at least two different forms of action in this setting. (1) Glucocorticoids prevent the formation of the ISG3 complex and thus prevent activation of the IFN-beta responsive genes. (2) Glucocorticoids bind to its receptor (GR) and this complex moves from the cytoplasm to the nucleus and binds to the glucocorticoid binding site of the IFNAR2 gene, if the individual has TT at SNP rs9984273. This seems to lead to repression seen as low expression of IFNAR2. In contrast, if the individual has CC or CT this binding does not efficiently take place resulting in higher expression of IFNAR2 than in TT individuals. The size and intensity of the glucocorticoid/GR complex and the IFNAR2 gene illustrate the relative magnitude of the phenomenon in CT vs TT patients

Both type I IFN and cortisol responses are needed in viral induced critical illness, but at different times, IFNs first and steroids later. In most people, both endogenous and exogenous steroids block the organ protective effects of IFN β on the endothelium, if there is no preceding type I IFN response. We have discovered a genetic polymorphism rs9984273 that prevents GR affecting IFNAR expression, but allows glucocorticoids to shut down STAT1-STAT2 type I IFN signaling. A schematic picture (Fig. 4) is illustrating our hypothesis based on this new finding and existing literature. This genetic polymorphism has a significant role in disease states, where both type I IFNs and steroids have an impact on mortality.

We found TT patients to have a trend for lower total STAT1 expression and statistically significantly less pSTAT1 than the CT patients. In contrast, TT patients had significantly more STAT2 than the CT patients, while no statistically significant differences in pSTAT2 were observed suggesting that interferon signaling differ between these genotypes. However, detailed picture regarding the dynamics is impossible to draw based on the level of STAT1/STAT2 expression, because in addition to STAT1/STAT2/IRF9 complex JAK can phosphorylate and initiate complexes of STAT1 homodimers [28, 29]. Moreover, type I IFNs have been reported to activate formation of STAT2:STAT3 heterodimers [30].

We detected faster decrease in IFN γ and IL-6 levels in patients with the minor allele (CT or CC) compared to those homozygous with the major allele (TT) in rs9984273 when glucocorticoids were administered. Lung specimens of TT patients had also significantly more STAT2 than the CT patients and glucocorticoids did not decrease its nuclear expression like in CT patients. It has been shown that unphosphorylated STAT2 upregulates IL-6 via co-operation of the NF-κB pathway and can do it perfectly well without STAT1 [31]. In this scenario, we speculate that the “extra” STAT2 in patients with the rs9984273 TT genotype maintains the high IL-6 and IFN γ levels via a comparable mechanism. This idea is also supported by the finding that there is more unphosphorylated STAT2 in samples of TT than CT patients.

SNP rs9984273 is a relatively common polymorphism according to available data. Approximately 45% of people with African origin, 34% of Caucasians and 10% of Asians carry the polymorphism (C allele instead of T) https://www.ncbi.nlm.nih.gov/snp/rs9984273#frequency_tab, which may explain the better resilience of Africa in the current SARS-CoV2 pandemic, as well as vulnerability of the Asian population concerning steroid use for the treatment of COVID-19 [32]. In the INTEREST trial, 10.8% of the patients were CC, 40.7% CT, and 48.4% TT. The associations between rs9984273 and mortality in patients with ARDS, as well as hospitalizations with COVID-19 are novel findings as no prior clinical significance has been reported with this polymorphism. We observed more vigorous upregulation of *MX1* in CC/CT than TT patients. As *MX1* is a key mediator of the interferon-induced antiviral response against a wide range of viruses this may contribute to the better outcome of CC/CT patients with COVID-19.

Our findings regarding the IFNAR2 expression are also in line with the public data. The Genotype-Tissue Expression (GTEx) portal V8 release for significant variant-gene pairs showed that rs9984273 acts as an expression quantitative trait locus (eQTL) for IFNAR2 expression in lung tissue (*P* = 9.1 × 10^–7^). The CC and CT genotypes correlated with higher IFNAR2 expression compared to TT genotype based on the lung tissue data from 515 individuals. In the same data, the SNP rs2236757 also correlated with IFNAR2 expression but to a lesser extent (*P* = 7.3 × 10^–5^). The SNP rs2236757 AA genotype seemed to correlate with low IFNAR2 expression compared to AG and GG genotypes [33]. Unfortunately, the data to assess the combined effect of these two SNPs to the IFNAR2 expression is not publicly available.

One may argue that patients receiving glucocorticoid treatment were more severely ill than those without glucocorticoids and that explains our findings. To exclude that possibility, we earlier performed propensity matching for a matched dataset (*n* = 98). In this dataset, disease severity by APACHE II and SOFA scores did not differ between those receiving IFN β alone or combined with glucocorticoids (see the supplement of ref [5]).

The role of IFNAR in COVID-19 outcome is obvious as contribution of a third SNP in IFNAR to COVID-19 severity is reported by Ma et al. [34]. They found that individuals with G allele in rs9976829 of IFNAR2 have 16% greater chance of COVID-19 infection compared with those without G. However, rs9976829 and rs9984273 found by us are only in a mild linkage disequilibrium with (*r*^2^ = 0.22, D’ = 0.97) in the European population. A correlation of A allele, but not G allele, of rs9976829 and C allele of rs9984273 is seen in the European population. Thus, combination of several SNP seems to contribute to the outcome of COVID-19.

Is there a problem, if IFNAR expression is too high? In this context, the recent findings of Malle et al. [35] are interesting as they have analyzed down syndrome (DS) patients (trisomia of chromosome 21) who have higher gene load for IFNAR as it is located in chromosome 21. DS patients have less frequent viral infections. However, while increased IFNAR expression initially causes hypersensitivity to type I IFNs, it triggers negative feedback. In light of these findings, IFN response seems to be endogenously regulated, if the response is too vigorous. We can speculate that also in normal individuals there is a regulatory mechanism as well, but CC/CT is not likely to cause effects of such magnitude as in DS patients with triplication of the IFNAR genes. It would be interesting to see, whether CC/CT and TT play any role in antiviral response of DS patients.

One limitation in our study is that we do not have data regarding which patients have viral induced ARDS vs bacterial, or both, or fungal in the INTEREST trial, and therefore, the results of INTEREST do not perfectly represent the situation in COVID-19. Exact microbial origin should be registered in future ARDS trials as interferon responses, specifically type 1 responses are somewhat differentially required between bacterial and viral infections. Moreover, the exact molecular mechanisms of the GR binding site in IFNAR2 and its possible additional effects to other reported SNPs in IFNAR2 require future investigations.

We envision that the genetic polymorphism SNP rs9984273 reported in this paper has evolved and become enriched during previous viral outbreaks and pandemics in humans. Furthermore, we conclude that this polymorphism may, at least in part, explain the continuous controversy in studies investigating the use of glucocorticoids for ARDS, especially those of viral origin where type I IFN activity is needed. In summary, we have discovered a novel polymorphism in IFNAR2 containing a GR binding motif that is associated with the IFNAR2 expression level, IFN β response, and outcome of ARDS/COVID-19. Our study strongly indicates that simultaneous administration of IFN β and glucocorticoids should be avoided especially for those patients with TT allele in SNP rs9984273.

## Supplementary Information

**Additional file 1.** Supplemental Figures.

## Data Availability

All data are available in the main text or the supplementary materials. Patient samples are not available.

## Abbreviations

APACHE: Acute physiology and chronic health evaluation
ARDS: Acute respiratory distress syndrome
GWAS: Genome-wide association study
GR: Glucocorticoid receptor
IFN: Interferon
IFNAR: Interferon receptor
IL: Interleukin
ISG: Interferon-stimulated genes
ISRE: Interferon-stimulated response element
SNP: Single-nucleotide polymorphism
