# Supplementary material for: Polymorphism in interferon alpha/beta receptor contributes to glucocorticoid response and outcome of ARDS and COVID-19
Source: Crit Care. 2023 Mar 16;27:112. doi: 10.1186/s13054-023-04388-8 (PMC10018638; doi:10.1186/s13054-023-04388-8)
Supplement: Supplementary file 1 — Additional file 1. Supplemental Figures. [file 13054_2023_4388_MOESM1_ESM.docx]

**Supplemental Figures**


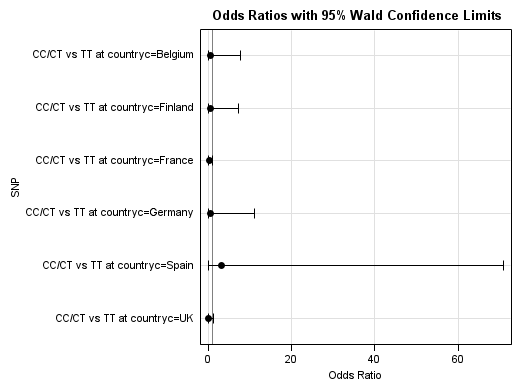


**Supplemental Fig. 1** Forest plot of the logistic regression analysis for day 28 mortality for effect of polymorphism (SNP) by country in the INTEREST trial. P= 0.7462 for interaction effect of polymorphism. (Italy left out of analysis since it did not have any deaths among these polymorphism classes)


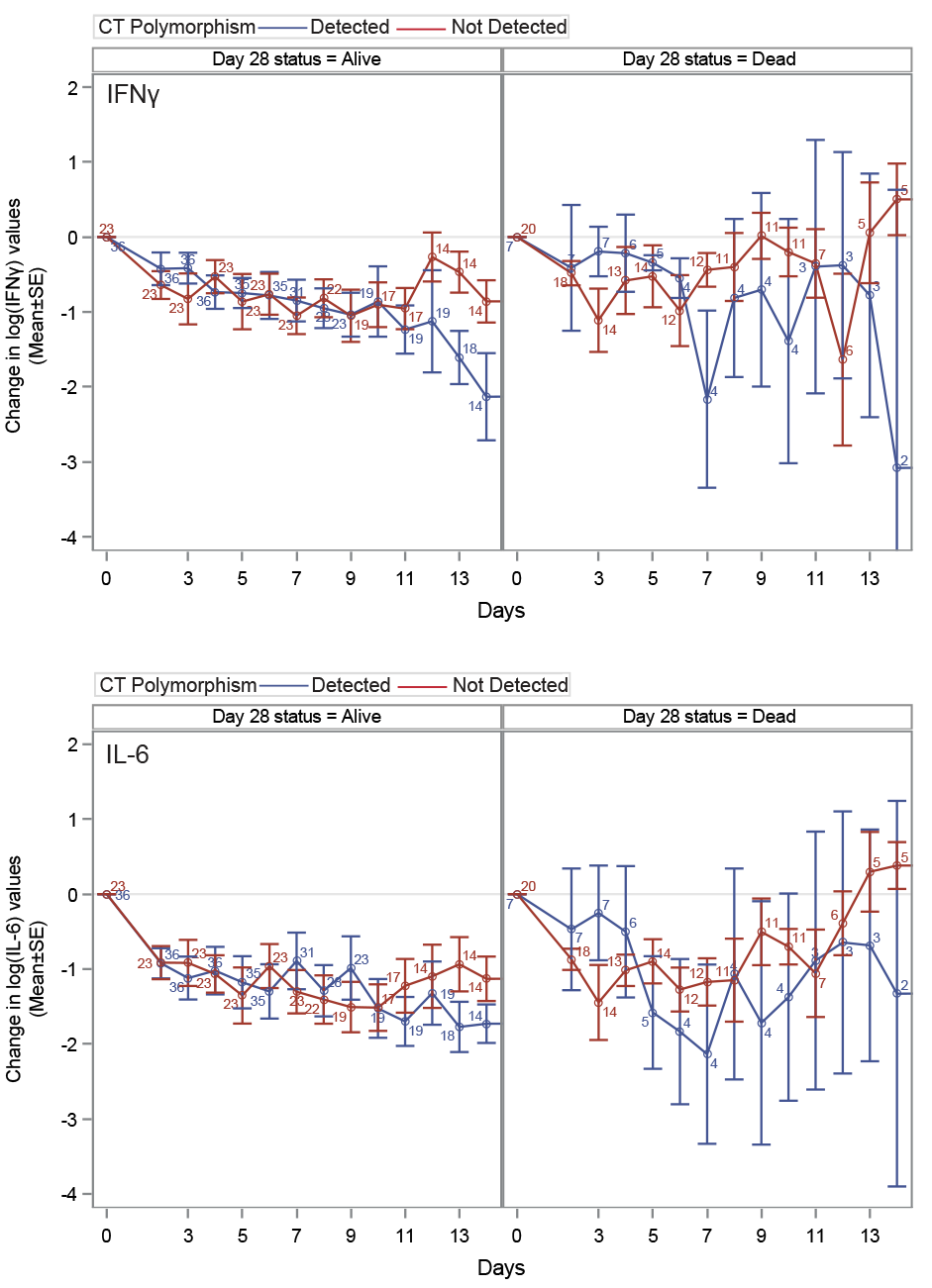
 **Supplemental Fig. 2** The logarithmic change in serum IFN γ and IL-6 levels according to the genetic background of rs9984273 in patients of the INTEREST trial with ARDS divided by mortality status at Day 28. Patients homozygous with the major allele (TT) compared to the minor allele (CT or CC) of rs9984273 show higher serum IFN γ and IL-6 levels when given glucocorticoids despite of the mortality status from Day 7 onwards (p<0.0001 for IFN γ and p=0.0018 for IL-6, for difference over timepoints in RM ANCOVA model when including only patients alive at Day 28). Numbers of the patients at each time point are indicated.

**Supplemental Fig. 3** Examples of pSTAT1 and pSTAT2 expression after 4-day culture in presence of IFN β. Expression of pSTAT1 and pSTAT2 was mainly detected in non-endothelial cells and was found both in the cytoplasm and nucleus (brown precipitate) as quantified in the summary panel (Figure 3E). The narrow arrows point to cells with main positivity in the nucleus, while the thick arrow points to the cell with clear positivity in the cytoplasm. Negative control stainings are shown in the insets. The Scale bar 20μm.
